# Curcumin improves expression of SCF/c-kit through attenuating oxidative stress and NF-κB activation in gastric tissues of diabetic gastroparesis rats

**DOI:** 10.1186/1758-5996-5-12

**Published:** 2013-03-01

**Authors:** Qi-Hui Jin, Hong-Xia Shen, Hui Wang, Qi-Yang Shou, Qiang Liu

**Affiliations:** 1 Department of Geriatric Medicine, The Second affiliated Hospital of Zhejiang University School of Medicine, Hangzhou, Zhejiang, China; 2 Department of Radiotherapy, The Second affiliated Hospital of Zhejiang University School of Medicine, Hangzhou, Zhejiang, China; 3 Department of Science and Education, Zhejiang Chinese Medical University, Hangzhou, Zhejiang, China; 4 Department of Animal experimental center, Zhejiang Chinese Medical University, Hangzhou, Zhejiang, China

**Keywords:** Curcumin, Diabetic gastroparesis, Oxidative stress, NF-κB, Stem cell factor/c-kit, Interstitial cells of Cajal

## Abstract

**Background:**

Diabetes mellitus is associated with many kinds of complications. Recent studies have shown that oxidative stress and inflammatory reactions have critical roles in the pathogenesis of diabetic gastroparesis. Curcumin is known to have antioxidant and anti-inflammatory properties. In the present study, we investigated the effect of curcumin on diabetic gastric motility in a Sprague Dawley rat model of type 1 diabetes mellitus.

**Methods:**

Male SD rats were divided into a control group, a control group receiving curcumin, a diabetic group, and a diabetic group receiving curcumin. Diabetes was induced by intraperitoneal injection of streptozotocin. Curcumin (150 mg/kg) was given intragastrically for 6 weeks, and blood glucose levels and body weights were measured. Stomachs were excised for analysis of gastric emptying rates, and levels of oxidative stress. NF-κB, I-κB, and stem cell factor (SCF)/c-kit protein levels were assessed by western blot analysis, while the apoptosis of interstitial cells of Cajal (ICCs) was assessed by TUNEL staining.

**Results:**

Curcumin-treated diabetic rats showed significantly improved gastric emptying rates [(59.4 ± 7.5)%] compared with diabetic rats [(44.3 ± 5.7)%], as well as decreased levels of MDA [21.4 ± 1.8 (nmol/mg) *vs* 27.9 ± 2.1 (nmol/mg)], and increased SOD activity [126.2 ± 8.8 (units/mg) *vs* 107.9 ± 7.5 (units/mg)]. On the other hand, the gastric emptying level in the control group was not significantly different from that in the control group receiving curcumin treatment. In addition, curcumin-treated diabetic rats showed significantly increased levels of SCF/c-kit protein in stomach tissues, inhibited I-κB degradation and NF-κB activation, and reduced ICC apoptosis index [(26.2 ± 4.1)% *vs* (47.5 ± 6.2)%], compared with the diabetic group.

**Conclusion:**

Curcumin treatment improved gastric emptying by blocking the production of oxidative stress, abolishing NF-κB signal transduction and enhancing expression of SCF/c-kit in rats with diabetic gastroparesis.

## Introduction

Diabetic gastroparesis is a disease of the digestive tract, defined as delayed emptying of a solid meal, and is seen in 30–50% of patients with type 1 or type 2 diabetes mellitus [1]. Patients often present upper gastrointestinal symptoms, such as early satiety, weight loss, abdominal bloating, abdominal discomfort, nausea, vomiting occur frequently, and impaired glycemic control, and the disease seriously affects patients’ quality of life.

Gastroparesis is increasingly being recognized as a significant health problem. Although gastroparesis affects many diabetics worldwide, not only are the treatment options very limited, but also the treatments that are available for diabetic gastropathy are frequently ineffective. These include medical therapy, gastric electrical stimulation, surgical therapy, and nutritional support. Medications for gastroparesis include metoclopramide, domperidone, cisapride, and erythromycin. While these agents have been used for treating gastroparesis, they have been reported to be of only limited efficacy, and many patients cannot tolerate them because of their side effects. In diabetic patients with refractory gastroparesis, high-frequency gastric electrical stimulation by a permanently implanted system significantly improved upper digestive tract symptoms and represents an alternative surgical therapy for patients. Surgical procedures, such as gastrectomy and antrectomy are the last option for treatment, being controversial and in need of more study [2]. In patients with severe gastroparesis but normal small intestinal motility, jejunostomy tube feeding may be applied. While this provides nutritional support, it does not cure gastroparesis [3]. Diabetic gastroparesis is generally best treated medically, and only patients with severe diabetic gastroparesis who have failed to respond to medical therapy should undergo other therapies. Thus, finding drugs that show efficacy in treating diabetic gastroparesis is necessary.

Diabetic gastroparesis is associated with a loss of interstitial cells of Cajal (ICCs) in both humans and model animals [4]. ICCs can be identified in tissue by labeling with antisera to the receptor tyrosine kinase kit, a protein expressed on ICCs. Kit and its ligand stem cell factor (SCF) are also important survival factors for ICCs [5]. The increased oxidative stress associated with diabetes can lead to loss of or damage to ICCs in mice, while oxidative stress leads to damage and loss of ICC networks and the development of delayed gastric emptying [6]. Diabetes results in increased oxidative stress and has an important role in the pathogenesis of diabetic complications [7]. Overproduction of reactive oxygen species (ROS) results in oxidative damage, including lipid peroxidation, protein oxidation, and DNA damage, which can lead to cell death. Furthermore, ROS are known to act as second messengers to activate transcription factors such as nuclear factor kappa B (NF-κB). ROS generated during stress in gastric tissue may trigger the activation of the NF-κB signaling cascade; indeed, gastric emptying in diabetic rats is associated with activation of NF-κB–mediated inflammation.

Curcumin (1,7-bis-(4-hydroxy-3-methoxyphenyl)-1, 6-heptadiene-3,5-dione) is the main active component of turmeric isolated from the plant curcuma longa L. Curcumin is a multifunctional molecule with significant regulatory effects on cancer [8], inflammation [9] and diabetic-related diseases such as diabetic retinopathy [10], diabetic nephropathy [11], and diabetes cognitive dysfunction [12]. Curcumin is a potent scavenger of reactive oxygen and nitrogen species such as hydroxyl radicals and nitrogen dioxide radicals [13]. Several studies have demonstrated that curcumin inhibits activation of NF-κB proinflammatory signaling pathways [14]. Meanwhile, NF-κB transcription factors regulate a number of important physiological processes, including inflammation and immune responses, cell growth and survival, so inhibition of NF-κB signaling represents a viable strategy for disease therapy [15]. However, there has been very little research on the potential for curcumin to treat diabetic gastroparesis, or on its pharmacological mechanism.

The goal of this study was to determine whether, in a Sprague Dawley (SD) rat model of type 1 diabetes mellitus, middle-dose [16] curcumin could protect ICCs in diabetic rats with delayed gastric emptying by reducing oxidative stress and inhibiting NF-κB activation, increasing SCF/Kit expression, and normalizing the delay in gastric emptying.

## Materials and methods

### Experimental rats and induction of diabetes

Male SD rats (8 weeks of age) were bred in the Center of Experimental Animals, Zhejiang Chinese Medical University, (Hangzhou, China), where a specific pathogen-free (SPF)-level laboratory has been authorized by the Zhejiang provincial government. All rats were housed under conditions of controlled humidity (50–60%). They were maintained under controlled light (12 h day/night cycle) with free access to water and rodent chow. All animal experiments were performed in consistency with the license from the Zhejiang Province Science and Technology Office (Hangzhou, China) and with approval from the animal ethics committee of Zhejiang Chinese Medical University. All experiments conformed to guidelines for ethical conduct in the care and use of animals. Every effort was made to minimize stress to the animals.

Rats fasted for 12 h were subjected to a single intraperitoneal injection of streptozotocin (STZ), 50 mg/kg, freshly dissolved in 100 mM sodium citrate buffer at pH 4.5. Age-matched normal rats received citrate buffer only. Development of diabetes was confirmed by fasting blood glucose (FBG) levels using a reagent kit (Roche, Shanghai, China). Rats with FBG levels higher than 11.1 mM (200 mg/dl) at 72 h after STZ injection were considered to be diabetic rats. Once the rats became diabetic, their glucose levels were measured daily. Experiments commenced 8 weeks after the development of diabetes to allow gastroparesis to develop.

### Experimental design

Eight weeks after the development of diabetes, animals were divided into four groups, i.e. normal control male rats (Group I), diabetic male rats (Group II), curcumin-supplemented diabetic male rats (Group III), and curcumin-supplemented control male rats (Group IV). Rats in groups III and IV (n = 10 each) were treated daily with curcumin (150 mg/kg; i.g.) for 6 weeks. Curcumin (purity > 95%) from Fusong County Natural Biotechnology Company Ltd. (Jilin, China), was suspended in 0.5% w/v sodium carboxymethyl-cellulose (CMC-Na) solution. Rats in groups I and II (n = 10 each) received 0.5% CMC-Na solution only. After treatment for 6 weeks, the animals were sacrificed under ethyl ether anesthesia, blood was collected by femoral vein bleeding and serum was separated. Stomachs were rapidly removed, and tissue samples collected from animals were stored at −80°C until processed for biochemical assays. Gastric emptying, oxidative stress, NF-κB activation, ICCs and SCF/Kit expression in stomachs were also studied at the end of the sixth week of treatment (14^th^ week of diabetes).

### Gastric emptying studies

The standard method for preparing a phenol red marker meal was employed as described previously [17]. Gastric emptying was determined using a modification of a previously reported procedure [18]. Rats were allowed free access to water until 3 h before gavage administration of curcumin. A solution of 0.1% (w/v) phenol red in aqueous sodium carboxymethylcellulose (1.5% w/v) was used as a test meal. Curcumin was given 1 h before the oral administration of the test meal. Twenty minutes after the administration of the test meal, the rats were sacrificed. The stomachs were then exposed by laparotomy and removed. Rats treated with the vehicle (saline, 0.9% sodium chloride solution, 0.2 mL) were sacrificed immediately after oral administration of the test meal and the phenol red content in the stomach was considered as the standard (100%). The removed stomachs were incised in 40 mL of NaOH solution (0.1 N) and the contents were dissolved. A 1-mL aliquot of the washing solutions was added to 2 mL of trichloroacetic acid (7.5% w/v) to precipitate proteins. After centrifugation (2500 × *g*) for 20 min, 1 ml of the supernatant was added to 1 ml of NaOH (1 N) to develop the maximum intensity of color. The absorbance at 558 nm of the solution was then measured using a spectrophotometer (U-1080; Hitachi Ltd., Tokyo, Japan). Gastric emptying was calculated according to the following formula:

$$\text{Gastric}\;\text{emptying}\;\left(\%\right)=\left(1–X/Y\right)\times 100,$$

where **X** is the absorbance of phenol red remaining in the stomach 20 min after phenol red administration and **Y** is the mean absorbance of phenol red recovered from the stomachs of control mice immediately after phenol red administration.

### Determination of MDA and SOD levels in smooth muscle of gastric antrum

The stomachs were excised, weighed, and immediately frozen at −70°C. Frozen tissue from each rat was homogenized in ice-cold phosphate buffer (KCl 140 mM, phosphate 20 mM, pH 7.4) and centrifuged at 3000 × *g* for 10 min. The content of malonaldehyde (MDA), an index for lipid peroxidation, was determined using the thiobarbituric acid (TBA) method with slight modification [19]. Briefly, 100 μl of tissue homogenate was mixed with 200 μl of work solution containing 0.37% TBA. The mixture was incubated at 100°C for 15 min and subsequently cooled. To extract MDA, 375 μl of N-butanol was added, followed by vortexing vigorously for 10 s. After centrifugation, the upper N-butanol layer was transferred to a glass tube. The absorbance of the butanol phase was measured at 532 nm. MDA content is expressed as nmol/mg protein. For measurement of SOD activity, 20 μl of tissue homogenate was mixed with 200 μl of reaction solution containing nitroblue tetrazolium chloride (NBT, 750 μM), and incubated for 20 min at 37°C. The absorbance at 560 nm was measured. Enzyme activity is expressed as units/g protein and 1 unit of enzyme was defined as the amount of enzyme required to inhibit the reduction of NBT by 50%.

### Semiquantitative RT-PCR measurement of SCF and c-kit

The proximal stomach (40–50 mg) was harvested, and the mucosa was removed by dissection. The tissue was mechanically homogenized under RNase-free conditions and dissociated with Tripure isolation reagent (Roche, Switzerland) on ice for 5 minutes, and total RNA was extracted according to the manufacturer’s instructions. Total RNA was quantified spectrophotometrically at 260 and 280 nm, with the A260/A280 ratio ranging from 1.8 to 2.0. RNA integrity was verified by agarose gel electrophoresis, followed by ethidium bromide staining, and RNA was used for immediate reverse transcription or stored at −80°C in RNase-free water. Two-step reverse transcriptase-polymerase chain reaction (RT-PCR) was performed using the Revertra ace-α- first strand cDNA synthesis kit (Tiangen Biotech, Beijing, China) and 2 × Taq PCR master mix (Tiangen Biotech), according to the manufacturer’s instructions. A fixed amount of RNA (0.5 μg) was reverse transcribed. Glyceraldehyde-3-phosphate dehydrogenase (GAPDH) was selected as an internal standard. All PCR primers were designed and synthesized by Biosune (Shanghai, China). The primer sequences and lengths of the PCR products are as follows: GAPDH, forward: 5^′^-GACAACTITG-GCATCGTGGA-3^′^, reverse: 5^′^-ATGCAGGGATGATGT-TCTGG-3^′^, 150 bp; SCF, forward: 5^′^-GGA CTT CAT GGT GGC ATC TG-3^′^, reverse: 5^′^-GCC CTT GTA AGA CTT GAC TG-3^′^, 285 bp; and c-kit, forward: 5^′^-GTG GTT AAA GGA AAC GCT CG-3^′^, reverse: 5^′^-CAT ACA TTT CAG CAG GTG CG-3^′^, 400 bp. Reaction conditions were optimized for each of the genes by varying the annealing temperature (58°C for GAPDH, 58°C for SCF, and 62°C for c-kit) and different numbers of PCR cycles [20]. The PCR products were separated on 2% agarose gels at 100 V for 30 minutes. Gel images were displayed on the liquid crystal display monitor of an ultraviolet transillumination PhotoDoc-Ite system equipped with a CCD camera (Bio-Rad Gel Doc 2000, Bio-Rad Laboratories, Hercules, CA) and preserved for intensity analysis. The relative mRNA levels of the selected genes were calculated as the ratio to GAPDH expression.

### Levels of SCF, c-Kit, IκB and NF-κB assessed by western blot analysis

About 50 mg of tissue was taken from the near proximal stomach. Tissues were cut into little pieces of about 0.25 cm^3^, homogenized, and dissociated in radio-immunoprecipitation assay lysis buffer (Hushang Biotechnology, Shanghai, China), containing 50 mM tris, 150 mM NaCl, 1% NP-40, 0.5% sodium deoxycholate, 1% sodium dodecyl sulfate, 1 mM PMSF, sodium orthovanadate, sodium fluoride, ethylenediaminetetraacetic acid, and leupeptin at 4°C for 30 minutes. Homogenates were centrifuged at 12 000 × *g* for 5 minutes at 4°C, and the supernatants were collected and used as total protein. Equal amounts of protein were electrophoresed by SDS-PAGE in 10% or 15% polyacrylamide gels. Proteins were transferred onto nitrocellulose membranes (Hushang Biotechnology) by semidry blotting. Membranes were blocked with Tris-buffered saline 0.1% Tween 20 (TBST) containing 5% milk for 60 minutes at room temperature, and then immuno-blotted with appropriately diluted primary antibodies at 4°C overnight. After washing three times with TBST, the blots were incubated with HRP-conjugated secondary antibody for 60 minutes at room temperature. Then, the complexes were visualized using an iChemi XR imaging system (WD9431A, Bomeike Biotechnology, China) with chemiluminescence reagents (Tigsun Biological Science Technology, Tianjin, China). Semi-quantification was performed using Quantity One software, version 4.6.2 (Bio-Rad). The following antibodies were used: anti-SCF, 1:200, c-kit, 1:200, anti-IκB and anti-NF-κB p65 and GAPDH, 1:500 (Hushang Biotechnology, Shanghai, China).

### Quantification of NF-κB -DNA binding by electrophoretic mobility shift assay

Nuclear extracts were prepared from stomach lysates as described previously [21]. NF-κB activation was determined by an electrophoretic mobility shift assay (EMSA), and nuclear translocation of NF-κB was assessed as described previously (GS009, Beyotime Institute of Biotechnology, Shanghai, China) [22]. For NF-κB binding reactions, 5 μg of nuclear protein extract was incubated at room temperature for 20 min with reaction buffer containing 20 mM HEPES, pH 7.9, 50 mM KCl, 0.1 mM EDTA, 1 mM DTT, 5% glycerol, 200 μg/mL BSA, and 2 μg of poly (dI-dC). Then, ^32^P-labeled double-stranded oligonucleotide (1 ng ≥1×105 cpm) containing the NF-κB binding consensus sequence (5^′^-GGCAACCTGGGGACTCTCCCTTT-3^′^) was added to the reaction mixture for an additional 10 min at room temperature. The reaction products were fractionated on a non-denaturing 6% polyacrylamide gel for 60 min at 350 V, which was then dried and subjected to autoradiography at −70°C overnight. For competition assays, excess oligonucleotide (100-fold molar excess) competitor was pre-incubated with nuclear extracts for 20 min at room temperature. A mutant NF-κB oligonucleotide (5^′^-GGCAACTGCTCACTCTCCCTTT-3^′^) was used for the competition assay. Signals were densitometrically analyzed.

### Double immunofluorohistochemistry with terminal deoxynucleotidyl transferase-mediated dUTP-biotin nick end labeling assay (TUNEL) and c-kit staining

Fluorescence immunohistochemistry for c-kit (using the antibody from Santa Cruz) and TUNEL staining were performed on 10-μm sections using a modification of the procedure published by the manufacturer of the TUNEL kit (TACS sTdT, Trevigen Inc, Gaithersburg, MD). TUNEL-positive cells were labeled with fluorescein (Ex 488, Em 505–550), c-kit-positive cells were labeled with Cy3 (Ex 543, Em 560–615), and nuclei were labeled with DAPI (Ex 364, Em 385–470). Tissue specimens were embedded in fluorescence mounting medium and investigated by confocal laser scanning microscopy [23]. Approximately 200 cells were counted per field, five fields were examined per slide and five slides were examined per group. The percentages of TUNEL- and c-kit-positive apoptotic cells were denoted as the apoptotic index (AI) (%).

### Statistical analysis

All data are presented as means ± SD. To compare data among groups of animals, one-way analysis of variance (one-way ANOVA) and Duncan comparisons were employed. All statistical tests were performed using SPSS for Windows, version 13.0 (SPSS Inc, Chicago, IL). Differences were considered statistically significant at *P* < 0.05.

## Results

### Effect of curcumin on body weight and blood glucose of diabetic rats

Plasma glucose levels were highly elevated in diabetic rats (Group II) [(322 ± 41) mg/dl] compared with those in normal control rats (Group I) [(98 ± 12) mg/dl]. There was a marked decline in the body weights of Group II rats [(298 ± 16) g] compared with age-matched Group I rats [(449 ± 21) g]. Chronic curcumin-treated diabetic rats (Group III) showed no significant improvements in body weight and no decline in plasma glucose levels compared with Group II rats [Group III, body weight (324 ± 19) g and glucose (302 ± 38) mg/dl] (Table 1).

**Table 1:** Blood glucose level and body weight in four groups

**Groups**	**Group I**	**Group II**	**Group III**	**Group IV**
**Blood glucose(mg/dl)**	98±12	322±41*	302±38*^#^	100±11
**Body weight(g)**	449±21	298±16*	324±19*	476±27

Group I=normal control rats; Group II=diabetic control rats; Group III=diabetic + curcumin treated rats (150 mg/kg); Group IV=normal + curcumin treated rats (150 mg/kg). Results are presented as mean ± SD. *P < 0.05 (compared to Group I), ^#^*P* >0.05 (compared to Group II).

### Effect of curcumin on gastric emptying

**Figure 1:**
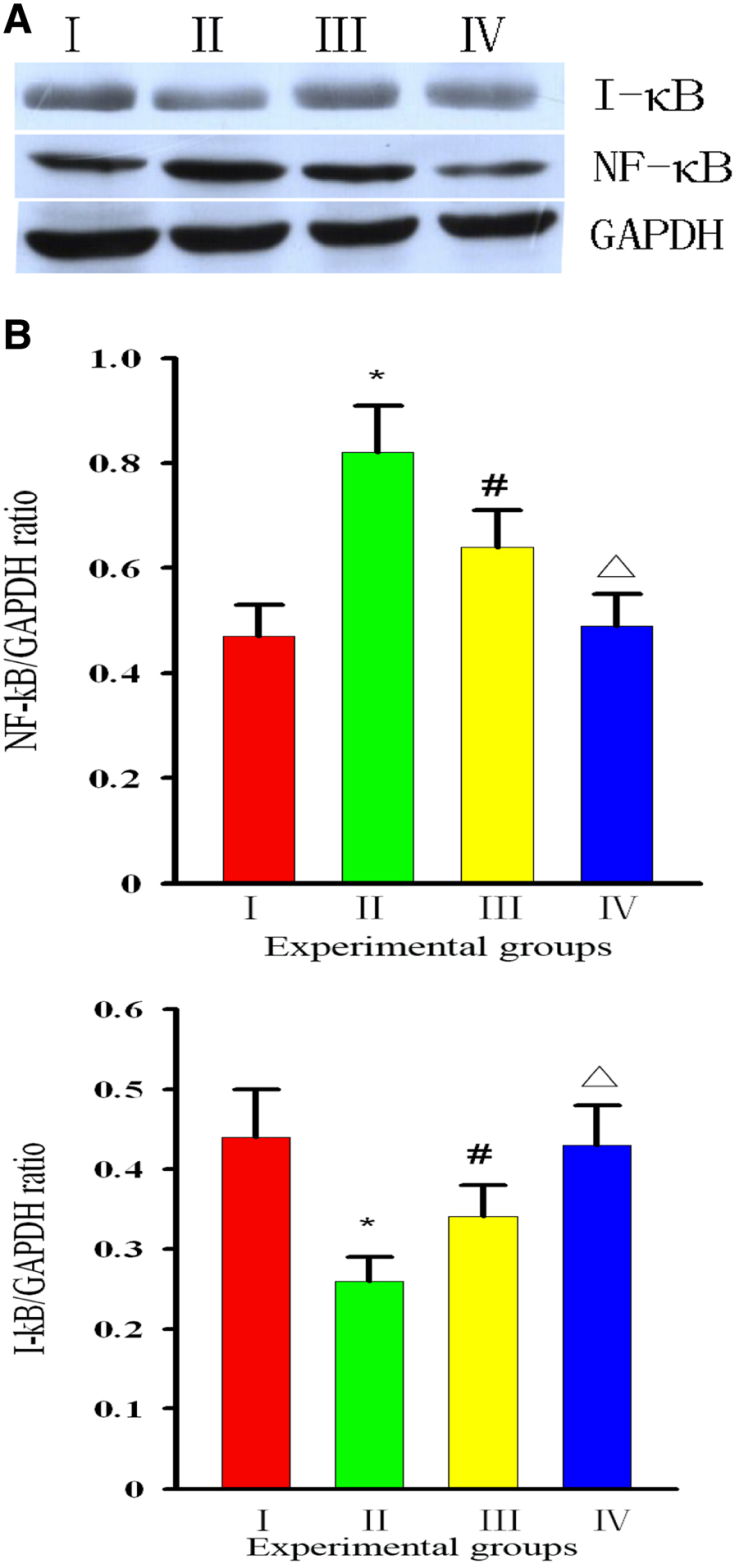
**Effects of curcumin on gastric emptying in diabetic rats.** Group I=normal control rats (n=10); **:** Group II=diabetic control rats (n=10); **:** Group III=diabetic + curcumin treated rats (n=10); **:** Group IV=normal + curcumin treated rats (n=10). All values are expressed as the mean ± SD. *p<0.01 (compared to Group I), # *p* <0.05 (compared to Group II), Δ*p* >0.05 (compared to Group I).

Gastric emptying was significantly delayed in SD rats that had had diabetes for 8 weeks. The amount of gastric emptying in Group II was 44.3 ± 5.7%, which was significantly lower than that in Group I [(76.2 ± 4.3)%, *P* < 0.01], and diabetic rats undergoing curcumin treatment [(Group III),; (59.4 ± 7.5)%, *P* < 0.05]. However, no significant difference was noted between Group I and Group IV rats [(73.3 ± 5.2%), *P* > 0.05] (Figure 1).

### Curcumin treatment decreased MDA formation, increases SOD activity

**Figure 2:**
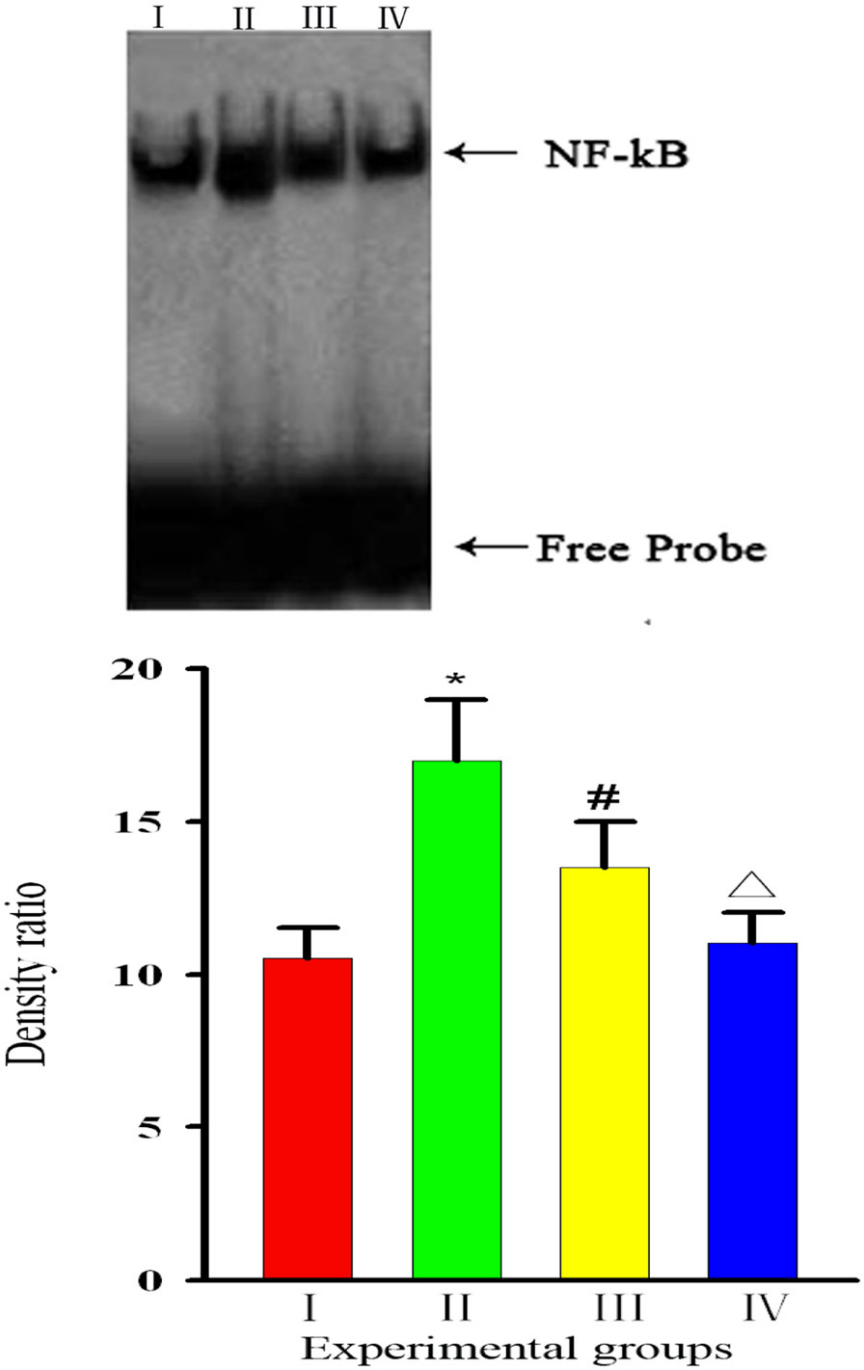
**Effects of curcumin on MDA and SOD in diabetic rats.** The levels of MDA was decreased after treated with curcumin, the levels of SOD was increased after treated with curcumin. **:** Group I=normal control rats (n=10); **:** Group II=diabetic control rats (n=10);Group III=diabetic + curcumin treated rats (n=10); **:** Group IV=normal + curcumin treated rats (n=10). All values are expressed as the mean ± SD.*p<0.01 ( compared to Group I), # *p* <0.01 (compared to Group II), Δ*p* >0.05 (compared to Group I).

We investigated oxidative stress using several methods because glucose-induced oxidative stress has been postulated to be a key mechanism in chronic diabetic complications. Diabetic rats exhibited increased MDA levels [Group I 15.7 ± 1.7 (nmol/mg) *vs* Group II 27.9 ± 2.1 (nmol/mg), *P* = 0.006] and decreased SOD levels [Group I 146.2 ± 9.3 (u/mg) *vs* Group II 107.9 ± 7.5 (u/mg), *P* = 0.008], a molecular marker of oxidative stress (Figure 2). Six weeks of curcumin treatment reduced the degree of MDA upregulation [Group III, 21.4 ± 1.8 (nmol/mg), *P* = 0.033 versus Group I and *P* = 0.004 versus Group II], and improved the SOD down-regulation [Group III 126.2 ± 8.8 (units/mg), *P* = 0.036 versus Group I and *P* = 0.001 versus Group II]. Curcumin significantly enhanced SOD activity and reduced MDA levels in stomach homogenates. Furthermore, no significant difference were noted between Group I and Group IV animals (*P* > 0.05; Figure 2).

### Curcumin increases SCF and c-kit protein levels

**Figure 3:**
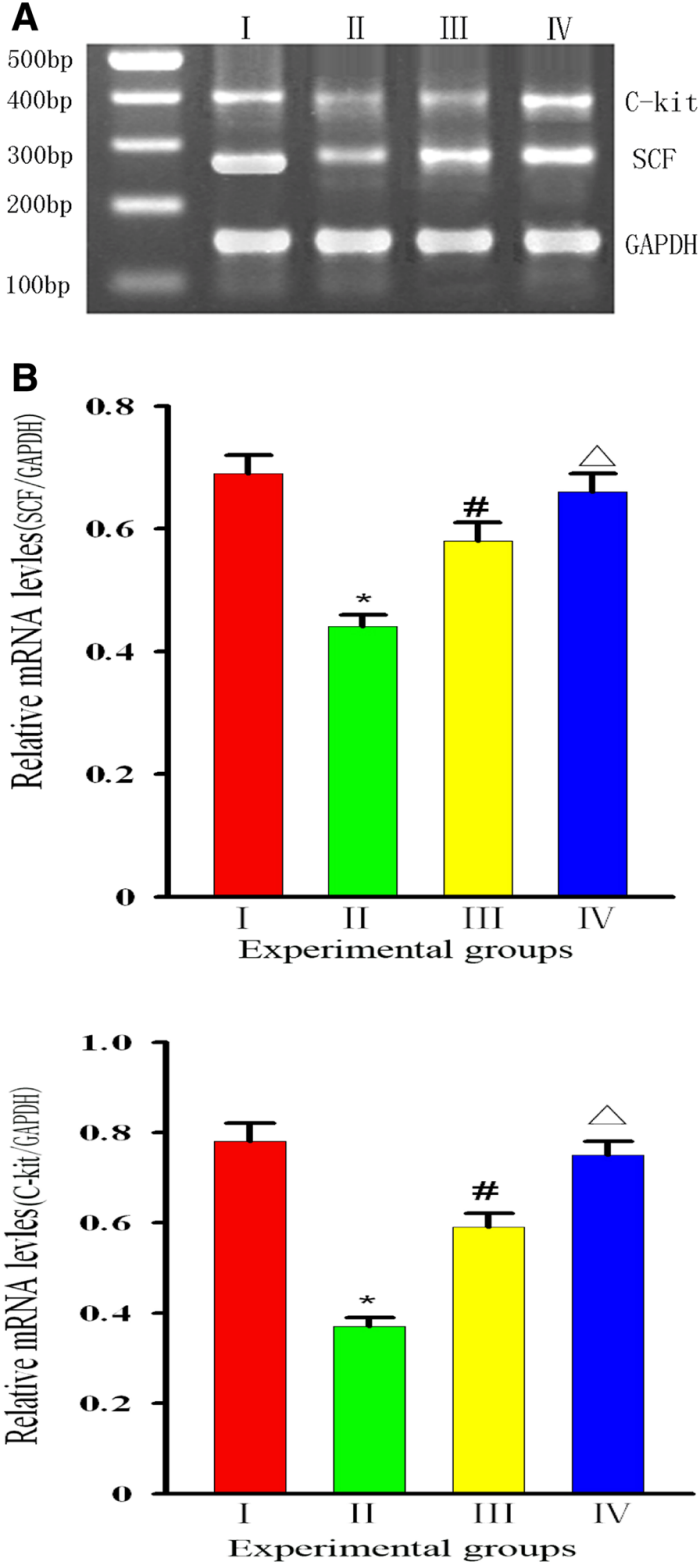
**Changes in SCF/GAPDH and C-kit/GAPDH by RT-PCR analysis. (A)** Representative images of gel electrophoresis of GAPDH,SCF, and c-kit in rats of each group.** (B) **Group levels of relative mRNA expression of SCF and c-kit, with products quantified by ratio to GAPDH. **:** Group I=normal control rats (n=10); **:** Group II=diabetic control rats (n=10); **:** Group III=diabetic + curcumin treated rats (n=10); **:** Group IV=normal + curcumin treated rats (n=10). All values are expressed as the mean ± SD. *p<0.01 (compared to Group I.), # *p* <0.05 (compared to Group II), Δ*p* >0.05 (compared to Group I).

**Figure 4:**
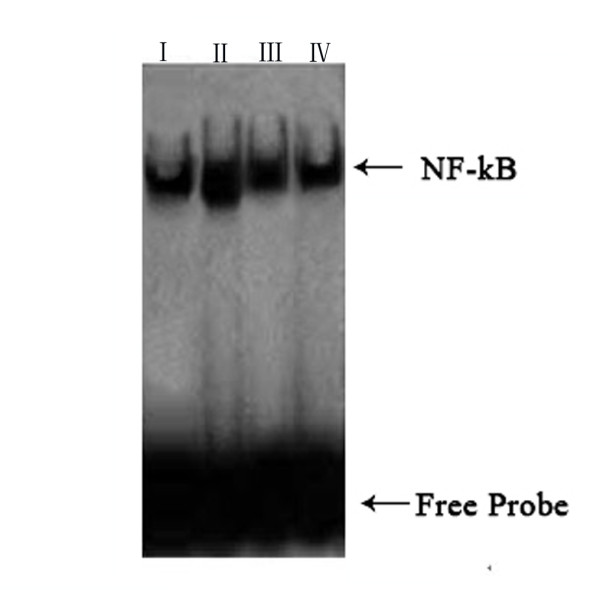
**Changes in SCF/GAPDH and C-kit/GAPDH by Western blot analysis. (A)** Representative protein band images of SCF, c-kit, and glyceraldehydes-3-phosphate dehydrogenase (GAPDH). **(B)** Group levels of relative SCF and c-kit protein expressed by ratio to GAPDH,respectively. **:** Group I=normal control rats (n=10); **:** Group II=diabetic control rats (n=10); **:** Group III=diabetic + curcumin treated rats (n=10); **:** Group IV=normal + curcumin treated rats (n=10). All values are expressed as the mean ± SD. *p<0.01 (compared to Group I), # *p* <0.01 (compared to Group II), Δ*p* >0.05 (compared to Group I).

Representative images of the RT-PCR analysis of SCF in the stomach tissues of rats in each group are shown in Figure 3**A**. Compared with Group I, the SCF was significantly lower in Group II [(0.44 ± 0.02) *vs* (0.69 ± 0.03), *P* = 0 .002], and a comparison of the values in Group III revealed that the SCF level in curcumin-treated rats was greater than that in Group II [(0.58 ± 0.04) *vs* (0.44 ± 0.03) *P* = 0 .003]. However, no differences were noted between Groups I and IV [(0.69 ± 0.03) *vs* (0.66 ± 0.03) *P* = 0.56] (Figure 3B). Western blot analysis (Figure 4A,B) revealed that the level of SCF in stomach tissues from Group II rats was significantly lower than those in Group I and III rats [ratios of (0.33 ± 0.02) *vs* (0.47 ± 0.03) and (0.41 ± 0.02), respectively, *P* < 0 .05)]. However, no significant difference was found between Group IV and Group I rats [(0.45 ± 0.03) *vs* (0.47 ± 0.03), *P* > 0.05)], suggesting that curcumin intervention could effectively restore local SCF protein levels in the stomach tissues of diabetic rats.

RT-PCR analysis showed that the expression levels for c-kit in stomach tissues were 0.78 ± 0.04, 0.37 ± 0.02, and 0.59 ± 0.03 for Groups I, II and III, respectively. The c-kit expression levels in Groups II and III rats were lower than that in Group I rats. However, a comparison of Groups II and III revealed that the c-kit level in curcumin-treated rats was greater than that in non-treated diabetic rats (*P* < 0.05, Figure 3B). Similar outcomes were demonstrated by western blot analysis (Figure 4B). The levels of c-kit protein (ratios of 0.45 ± 0.03, 0.21 ± 0.02 and 0.34 ± 0.02, respectively, for Groups I, II and III) were much greater in Group III than in Group II (*P* < 0 .05, Figure 4B), and the level in Group III was lower than those in Groups I and IV. Both analyses indicated that 6 weeks of curcumin intervention could significantly restore the decrease in c-kit levels in diabetic rats.

### Curcumin inhibits I-κB degradation and NF-κB activation

**Figure 5:**
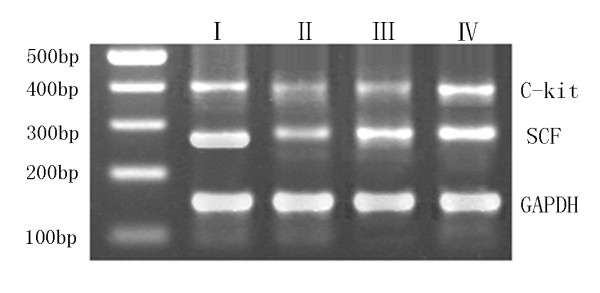
**Effect of curcumin on I-κB and NF-κB. (A)** the bar graph was clear reflection of the level of I-κB and NF-κB among four groups.** (B) ** the level of I-κB/GAPDH was decreased in Group II,but increased by curcumin. I**:** Group I=normal control rats (n=10); II**:** Group II=diabetic control rats (n=10); III**:** Group III=diabetic + curcumin treated rats (n=10); IV**:** Group IV=normal + curcumin treated rats (n=10). All values are expressed as the mean ± SD.*p<0.01 (compared to Group I), # *p* <0.01 (compared to Group II), Δ*p* >0.05 (compared to Group I).

**Figure 6:**
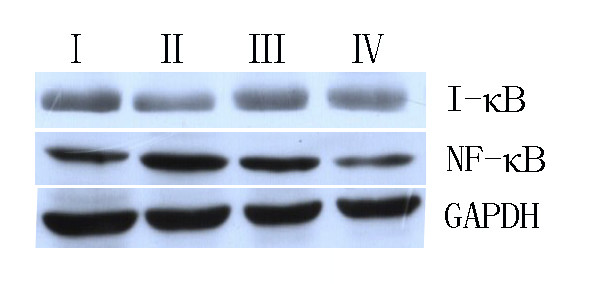
**Effect of curcumin on NF-κB activation.** Nuclear extracts were prepared and assayed for NF-κB -DNA binding activation by EMSA. The graph was clear reflection of the NF-κB -DNA binding activation among four groups. the level of NF-κB was increased in Group,but decreased by curcumin. Bars represent the mean ± SD from four different groups. **:** Group I=normal control rats (n=10); **:** Group II=diabetic control rats (n=10); **:** Group III=diabetic + curcumin treated rats (n=10); **:** Group IV=normal + curcumin treated rats (n=10). All values are expressed as the mean ± SD. *p<0.01 (compared to Group I), # *p* <0.01 (compared to Group II), Δ*p* >0.05 (compared to Group I).

Reports indicate that increased and chronic oxidative stress can activate or perturb NF-κB activity [24]. We investigated whether curcumin could result in activation of the NF-κB signaling pathway. To do this, we measured the levels of NF-κB in the presence or absence of curcumin to identify whether NF-κB was activated. First, we evaluated the protein levels of I-κB, to which inactivated NF-κB binds. We found that the level of I-κB in Group I rats was higher than that in Group II rats, while the level in Group III rats was lower than that in Group II rats (p < 0.01), but higher than the levels in Groups I and IV rats (p < 0.05). Then, we examined whether curcumin could block NF-κB. We found that diabetes (Group II) led to an increase in NF-κB levels [Group I 75.5 ± 7.7 (pg/ml) *vs* Group II 102.8 ± 12.4 (pg/ml), *P* = 0.001], while treatment with curcumin [Group III 91.3 ± 8.9 (pg/ml)] prevented this effect. Based on this finding, we suggested that curcumin suppresses activation of NF-κB [Group IV 70.9 ± 8.1 (pg/ml), *P* = 0.026] (Figure 5). We examined whether curcumin could block NF-κB translocation into the nucleus, because nuclear translocation is recognized as a cell reaction to ROS stimulation and seems to correlate with NF-κB-mediated transcriptional activation. To assess this, we measured the level of NF-κB in the nucleus by EMSA. Development of diabetes induced an increase in NF-κB levels in the nucleus, while treatment with curcumin prevented this effect (Figure 6). Thus, we suggested that curcumin not only indirectly suppresses activation of NF-κB–DNA binding, but that it also suppresses activation upstream of I-κB.

### Curcumin reduces apoptosis of ICCs and promotes their proliferation in stomach tissues

**Figure 7:**
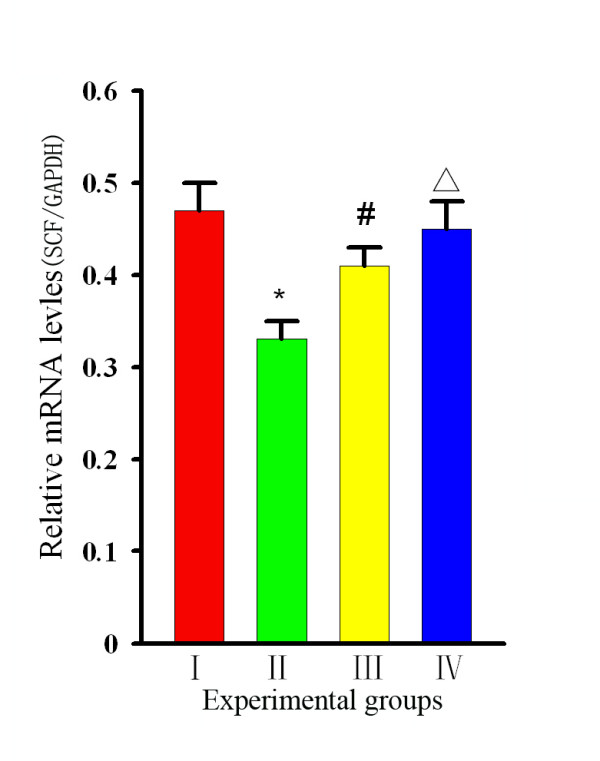
**Double Immunofluorohistochemistry With TUNEL and c-Kit in stomach tissues(×400 times). A:** Group I=normal control rats; **B**. Group IV=normal + curcumin treated rats **C**. Group III=diabetic + curcumin treated rats. **D**. Group II=diabetic control rats; Representative images of TUNEL positive and c-kit positive ICC from sections of stomach tissues of diabetic rats.Red were c-kit positive cells in tissues. Green were apoptotic cells in tissues.Yellow were TUNEL positive and c-kit positive apoptotoc ICCs.Numbers of TUNEL positive and c-kit positive apoptotic ICCs(yellow stain)expressed as the % of total number of ICCs in the circular muscle layer of each section. Yellow-stained ICCs are undergoing apoptosis.

To further analyze the chemopreventive action of curcumin on diabetic gastroparesis, we examined its effect on apoptosis in diabetic stomach sections by double immunofluorohistochemistry. Figure 7 shows representative images of apoptosis in stomach tissue sections from rats of all four groups. The chromogen-generated yellow stain is an indication of apoptotic ICCs. It should be noted that in almost every case the yellow stain overlaps the condensed chromatin of apoptotic bodies, thus confirming that the results of the double immunofluorohistochemistry assay correlate well with the morphological appearance of apoptosis. The rate of apoptosis was generally very low in Group I rats. Typically, in sections from diabetic rats, we found an average of 80–100 apoptotic cells in a field of about 200 cells. This number decreased to 40–50 stained cells in the curcumin-treated rats. As seen in Table 2, the proportion of apoptotic ICCs in the stomachs [AI:(47.5 ± 6.2)%] of Group II rats was markedly higher than that in the stomachs of Group I rats [AI:(6.4 ± 1.8)%]. This effect of diabetes was mostly reversed by curcumin [(26.2 ± 4.1)% for Group III], with a difference that was statistically significant (*P* < 0.05). However, no significant difference in AI was noted between Group I and IV rats [AI for Group IV: (7.1 ± 2.4)%] (*P* > 0.05).

**Table 2:** Apoptotic value of ICCs in stomach tissue sections in four groups

**Groups**	**Apoptotic indexa(AI)(%)**
Group I	6.4±1.8*
Group II	47.5±6.2
Group III	26.2±4.1^#Δ^
Group IV	7.1±2.4*

Group I=normal control rats; Group II=diabetic control rats; Group III=diabetic +curcumin treated rats (150 mg/kg); Group IV=normal + curcumin treated rats (150 mg/kg).* *P*<0.001, significant difference compared to Group II; # *P*<0.01, significant difference Compared to Group II. Δ *P*<0.05 significant difference compared to Group I and Group IV.

## Discussion

Numerous recent studies have demonstrated the ability of curcumin to halt or prevent certain types of cancer, decrease inflammation, and improve cardiovascular health. However, very few studies have examined its ability to protect against diabetic gastroparesis. In our study, we demonstrated that supplementation with curcumin attenuated oxidative stress and NF-κB activation, and prevented the down-regulation of SCF/c-kit protein level in diabetic rats. Our result suggests that the increased gastric dysfunction in diabetic rats can be prevented by curcumin treatment.

Data presented in this study show that SCF/c-kit is down-regulated after the development of diabetes and the concurrent increase in the level of oxidative stress. This down-regulation of SCF/c-kit was lost in all rats that went on to develop delayed gastric emptying and maintained in all rats that did not develop delayed gastric emptying. Failure to maintain down-regulation of SCF/c-kit and develop delayed gastric emptying was associated in all rats with high levels of markers of oxidative stress and NF-κB. In the curcumin-treated rats, ICCs were protected, suggesting that the maintenance of normal gastric function requires decreased ROS, restrained NF-κB activation and improved SCF/Kit expression (*e.g.* restored to normal levels).

Diabetes is a pathologic condition, and the morbidity and mortality associated with diabetes are the result of myriad complications related to the disease. Oxidative stress plays an important role in the etiology of the disease, and is considered to be the main factor leading to the development of diabetic complications and tissue injury [25]. Diabetes is associated with increased oxidative stress [26]. Oxidative stress mediates many of the deleterious effects of diabetes on organ function, and induces not only gastric mucosal injury, but also gastric motility dysfunction, such as diabetic gastroparesis [7]. Gastroparesis is thought to be caused by ROS-induced damage to the networks formed by ICCs [27]. Moreover, antioxidants can reverse diabetic gastroparesis in NOD mice [6]. ICCs in normal tissues are resistant to oxidative stress caused, for example, by hyperglycemia [28], but they can become vulnerable to glycemic stress when antioxidant defenses are compromised [6]. The results of our study show the potential mechanisms by which oxidative stress induces gastric motility dysfunction in diabetic rats. Diabetic rats exhibited slower gastric emptying together with increased MDA levels and decreased SOD levels compared with normal rats, with a tight correlation between high levels of MDA and the presence of delayed gastric emptying. Why does oxidative stress damage stomach function? ROS might inflict direct damage to vital cell constituents such as lipids, proteins and DNA, but also modulate patterns of gene expression through functional alterations of transcription factors such as NF-κB [29]. First, mature ICCs are easily damaged under conditions of oxidative stress [6]. ROS are a crucial regulator of cellular signal transduction and energy transmission, and a disturbance of the balance between ROS-generating and ROS-scavenging capabilities might lead to cell damage. Oxidative stress is now recognized as a stimulator of cell responses such as apoptosis [30]. A growing number of studies have described that ROS can activate inflammasomes in cells, leading to increased production of TNF-α, IL-1, IL-6 and IL-18 [31]. All sorts of inflammatory factors may directly damage ICCs. We examined the effect of curcumin on ROS formation in stomach tissues. Our finding that ROS content was significantly greater in Group II animals than in Group III animals suggests that curcumin attenuates diabetes-induced ROS formation. Our results also revealed that curcumin is a potent inhibitor of ROS-induced apoptosis. NF-κB is known to be a key factor in up-regulating inflammatory cytokines. NF-κB activation enhances the transcription of pro-inflammatory cytokines, and these cytokines in turn activate NF-κB [14]. A perturbation of NF-κB distribution promoted inflammatory mediator-mediated ICC apoptosis. NF-κB is normally located in cytoplasm where it binds I-κB to form an inactive complex. The phosphorylation and subsequent degradation of I-κB result in NF-κB activation. We found that the NF-κB level in the cytoplasm was higher in diabetic rats than in control rats. Activated NF-κB migrates into the nucleus, and causes the expression of inflammatory cytokines. A comparison of the nuclear NF-κB levels in Group II and III rats revealed that nuclear localization of NF-κB is markedly inhibited by curcumin. We also found that I-κB is degraded in the stomach smooth muscle of diabetic rats. Activation of NF-κB was induced by phosphorylation of the inhibitor IkB, in response to diverse stimuli including ROS, which leads to its degradation and results in unmasking of nuclear localization signals that allow NF-κB to be translocated into the cell nucleus [32]. ROS can directly activate NF-κB by degrading or modifying I-κB in the cytoplasmic NF-κB–IκB complex [33]. The results of this study show that, in rats with experimentally induced gastroparesis, proteolysis of IκB results in activation and nuclear translocation of NF-κB, and that this was accompanied by ROS up-regulation. Previously, various authors have reported that curcumin is a potent inhibitor of transcription factors [34,35], and that the suppression of NF-κB activation by curcumin results, in turn, in a down-regulation of ROS and inflammation [36].

It was suggested that loss of ICCs might have a major role in the pathogenesis of diabetic gastroparesis [37], and that the degree of ICC loss was in proportion to the severity of symptoms or changes in gastric emptying. Curcumin neutralized diabetes-induced oxidative stress, reduced MDA levels, improved SOD levels, restored NF-κB activity, and inhibited inflammatory mediator production, thereby minimizing diabetes-induced ICC apoptosis. All of these observations point toward a crosstalk between a ROS–NF-κB–inflammation mediator in deciding the fate of ICC cells in a high glucose micro-environment, and the intervening role of curcumin.

Reduced SCF levels in the stomachs of diabetic mice have been reported [38]. Because SCF/c-Kit signaling is important for the maintenance of ICC phenotypes, proliferation, and differentiation [25], we investigated whether ICCs could regenerate after impairment in diabetic rats following curcumin treatment. Furthermore, the kit ligand SCF was mainly observed in the smooth muscle cells (SMCs), which are located close to ICCs. In the proximal stomach, intramuscular ICC rather than myenteric ICC are the main subtypes of ICCs that give rise to gastric slow waves and act as the dominant pacemaker cells. Previous studies have shown that SCF is important for maintaining a steady level of ICCs in stomach tissues. SCF is produced by SMCs in the long-term maintenance of ICCs, while ICC depletion in diabetes is accompanied by smooth-muscle atrophy and reduced SCF levels [39]. Exogenous SCF can partially reverse the pathological changes in ICCs in diabetic mice, because SCF is necessary for differentiation of precursors into ICCs [38]. In our study, we found the SCF/c-kit levels and ICC numbers were clearly declined in diabetic gastroparesis rats compared with control rats. Indeed, the loss of SCF might be responsible for the loss of ICCs. The levels of mRNA expression for SCF and c-kit were lower in the stomach tissues of diabetic rats than non-diabetic rats, and decrease was significantly reversed by the curcumin intervention. These findings showed that curcumin improves SCF/c-kit levels in the stomach tissues of diabetic rats. These results also indicate that the fate of ICCs in diabetes depends on SCF/c-kit from SMCs, but probably not that from myenteric neurons [38]. No differences in fasting blood glucose levels and body weight were noted before and after treatment in the same groups of rats, indicating that the pathologic ICCs changes can be attributed to a deficiency of endogenous SCF/c-kit, and not related to hyperglycemia. We showed that ICC loss owing to diabetes involves reduced expression of SCF/c-kit and a critical differentiation and survival factor for ICCs. Developmental studies have demonstrated that c-kit^+^ mesenchymal precursor cells appear to generate ICCs and the longitudinal muscle layer [39]. Cell fate decisions between becoming an ICC or a SMC depend on SCF/c-kit signaling. We found that a loss or lack of SCF and c-kit ligand could lead to the apoptosis of ICCs, but when Group II rats were treated with curcumin, we observed an increase in SCF/c-kit concentration in the micro-environment promoting ICC transdifferentiation back into a normal phenotype. Our results suggest that curcumin could aid in the restoration of the SCF/c-kit signaling pathway, which is essential for ICC phenotype restoration and functional recovery. These results reinforce the results of previous studies showing that ICCs displaying distinctive plasticity can be controlled by changing the level of SCF/c-kit signaling.

Overall, our results show that curcumin has an effect on anti-oxidation and free radical removal. We further show that curcumin reduces activation of NF-κB via inhibition of oxidative stress. In addition, our results suggest that curcumin promotes the expression of SCF/c-kit. As a result, we have a better understanding of the molecular mechanism by which curcumin protects ICCs, namely, via blocking of oxidative stress, inhibition of NF-κB activation and an enhancement of SCF/c-kit expression.

## Conclusion

We suggest that curcumin can have a remarkable protective effect on ICCs and a therapeutic effect on gastric emptying dysfunction in diabetic rats.

## Competing interests

The authors declare that they have no competing interests.

## Authors’ contributions

WH, SHX and SQY carried out the experimental work, biochemical analysis, and statistical analysis, and performed interpretation and discussion of results related to their part of the work. JQH and LQ designed and planned the study, and drafted and revised the manuscript. All authors read and approved the final manuscript.
